# Endoscopic ultrasound–guided fine-needle biopsy as a novel approach for risk stratification of pancreatic cystic lesions

**DOI:** 10.1016/j.igie.2025.01.009

**Published:** 2025-01-17

**Authors:** Ghazal Hashemipour Moussavi, Maria F. Gomez, Jennifer Permuth, Aamir Dam, Anjuli K. Luthra, Luis Pena, Mark Friedman, Saraswathi Cappelle, Barbara Centeno, Shaffer R.S. Mok

**Affiliations:** 1 University of South Florida Morsani College of Medicine, Department of Gastrointestinal Oncology, Tampa, Florida; 2 H. Lee Moffitt Cancer Center and Research Institute, Department of Cancer Epidemiology, Tampa, Florida; 3 H. Lee Moffitt Cancer Center and Research Institute, Tampa, Florida

## Abstract

**Background and Aims:**

Unlike fine-needle biopsy (FNB), fine-needle aspiration (FNA) is limited by its inability to preserve histopathology. We compared the diagnostic yield of endoscopic ultrasound (EUS)–guided FNA and EUS-FNB for pancreatic cystic lesions (PCLs), hypothesizing that EUS-FNB would frequently provide a tissue diagnosis.

**Methods:**

A single-center retrospective cohort study was conducted from 2022 to 2023 on patients with PCLs who underwent either EUS-FNA or EUS-FNB. We compared cyst characteristics and pathology reports to assess diagnostic yield. The relative risk (RR) was calculated to compare FNB’s probability of identifying intraductal papillary mucinous neoplasm (IPMN) grade compared with FNA.

**Results:**

A total of 130 PCLs were identified (FNA: n = 34; FNB: n = 96). Patients had a mean age of 68 ± 12 years, and 49% were women. Sex, age, cyst size, location, and presence of pancreatic ductal dilation did not differ significantly between FNA and FNB groups. FNB showed a significantly higher diagnostic yield compared with FNA (81% vs 62%; *P* = .02), with better performance in identifying an IPMN grade compared with FNA (RR = 1.92; *P* = .013).

**Conclusions:**

Our results reflect one of the first studies, to our knowledge, to consider the diagnostic role of FNB in evaluating PCLs. These preliminary results suggest that FNB may have a potential for high diagnostic performance in PCLs and warrant further exploration using a larger cohort and randomized controlled trials.

The increased use of imaging studies such as computed tomography (CT) and magnetic resonance imaging (MRI) has raised the incidental detection of pancreatic cystic lesions (PCLs).^1^^,^^2^ Identification of PCLs on imaging warrants further risk stratification of the cyst to guide management owing to the potential malignancy of some PCLs. One classification for PCLs is mucinous versus nonmucinous. Mucinous types such as intraductal papillary mucinous neoplasm (IPMN), particularly those with main duct involvement, have a higher risk of malignancy, with rates of 33% to 60% in surgical series.^3^

Besides imaging, diagnostic standards for PCLs currently involve endoscopic ultrasound (EUS) with fine-needle aspiration (FNA) of cyst fluid for carcinoembryonic antigen (CEA) and cytology.^2^ Current Fukuoka and American College of Gastroenterology (ACG) guidelines recommend EUS ± FNA for symptomatic lesions, cyst ≥3 cm, main duct changes, or main duct diameter >5 mm.^4^^,^^5^

The 2 methods of minimally invasive tissue acquisition for pancreatic solid lesions are EUS-guided FNA and fine-needle biopsy (FNB). In a meta-analysis by Facciorusso et al,^6^ for solid pancreatic lesions no specific EUS-guided tissue sampling (either FNA or FNB) technique was found to be superior when considering diagnostic accuracy and sample adequacy. In the case of PCLs, current guidelines recommend the use of FNA.^4^^,^^5^ In a meta-analysis of 18 studies with a total of 1438 patients, EUS-FNA identified pancreatic cystic mucinous neoplasm via cytology with 54% sensitivity and 93% specificity.^7^ This moderate sensitivity but high specificity may be due in part to the poor cellularity in pancreatic cyst cytologic samples (34% suitability in 1 study) that hinders evaluation.^4^

Besides cytology, sensitivity and specificity of FNA for categorizing a cyst can be improved by analyzing the cyst fluid for CEA, glucose, amylase, and genetic testing. A CEA with a cutoff of 192 ng/mL has 63% sensitivity and 88% specificity.^7^ Although not routinely used in practice, intracystic glucose of <50 mg/dL also has shown high sensitivity in diagnosing mucinous cystic neoplasm and intracystic amylase of <250 IU/L in excluding pseudocysts.^4^^,^^8^ More costly options, such as somatic genetic sequencing of cyst fluid, are other avenues for analysis, with DNA mutation analysis showing promise in identifying IPMNs and mucinous cystic neoplasms.^4^

Although tissue acquisition with FNA is able to assess for cytology, it is limited by the inability to obtain histologic architecture and subsequent immunohistochemical staining for high-risk features.^9^ To overcome the limitations of FNA, biopsy can be used to obtain histologic tissue samples for improved diagnostic accuracy. Thus far in the literature, biopsy of PCLs has been reported by the use of through-the-needle microforceps, which are passed through a standard 19-gauge EUS-FNA needle.^10^ Though promising, it is not recommended by the ACG guidelines^4^ published in 2018 owing to lack of robust evidence. However, since then, multiple studies have been conducted, and a meta-analysis in 2020 found through-the-needle biopsy to have 85.3% sample adequacy and significantly better performance than FNA in sample adequacy (*P* = .004) and diagnostic accuracy (*P* = .01). Risks associated with the procedure were bleeding (4%) and pancreatitis (2%).^10^ Limitations of studies on microforceps include lack of randomized studies and potential biases in diagnostic accuracy given that a small number of patients underwent surgery.^10^

Although through-the-needle biopsy is promising, it is not widely used in the clinical setting. One type of fine-needle biopsy used in sampling solid lesions is a Franseen-tip needle, the utility of which has not been demonstrated in the literature for PCLs. The present study aimed to explore the use of this FNB method in PCLs. In this single-institution retrospective study, we compared the diagnostic yield from EUS-FNA and EUS-FNB for PCLs. We hypothesized that EUS-FNB would yield a tissue diagnosis more often than EUS-FNA in a population of PCLs.

## Methods

This was a retrospective cohort study of patients with PCLs who underwent evaluation with either EUS-FNA or EUS-FNB from 2022 to 2023 at an National Cancer Institute–designated comprehensive cancer center. The study was approved by the institutional review board (IRB 22156, registered September 2, 2022) and considering that it was retrospective, a waiver of informed consent was granted. Nonetheless, per hospital policy, before all procedures, informed consent was obtained.

The inclusion criteria entailed patients ≥18 years old with evidence of PCL from CT or MRI who underwent subsequent cyst examination with either EUS-FNA or EUS-FNB. We excluded patients who underwent EUS but for whom no FNB or FNA was attempted and patients for whom no pathology report was available. In the case that a patient had multiple pancreatic cysts requiring analysis during 1 procedure session, only 1 method (either FNA or FNB) was used for sampling. For patients with multiple pancreatic cysts, we excluded EUS information about cysts that were not sampled; thus, our data reflect only cysts that were sampled.

Using the inclusion and exclusion criteria, we identified patients from the hospital’s registration database. We reviewed their electronic medical record for procedural information, demographics, cyst characteristics as determined by EUS, pathology and cytology report, medical history, and adverse events (AEs) within 7 days following the procedure.^11^ If a patient with multiple cysts had more than 1 cyst sampled, all sampled cysts were included. Demographic information was analyzed based on unique patients rather than cyst count. The choice of whether a cyst was examined by FNA (standard non–Franseen-tip needle) or FNB (Franseen-tip needles) using full suction, the technique used, and the needle gauge were at the discretion of the 3 board-certified advanced endoscopy-trained gastroenterologists performing the procedure (who perform at least 350 annually), patient choice, and schedule availability. Some cases involved advanced endoscopy trainees. The FNB and FNA typically involved the use of a 19-gauge or 22-gauge needle under ultrasound guidance, with transduodenal approach for head and uncinate PCLs and transgastric approach for PCLs in the body or tail of the pancreas. One pass was performed with the use of full suction and without stylet. Color Doppler imaging confirmed the absence of vascular structures before puncture. Prophylactic antibiotics were not administered unless the procedure was accompanied by confocal endomicroscopy, the result of which was not used in the present study.

Diagnostic yield was defined as any pathology report that yielded a report significant for categorizing a cyst and determining dysplasia of a cyst if applicable.^12^ Nondiagnostic reports included pathology reports that did not yield a diagnosis. Nondiagnostic reports were labeled by the pathologist as either nondiagnostic, scant cellularity, or samples with mostly contaminants. All samples were reviewed by a cytopathologist with expertise in pancreaticobiliary pathology and a dedicated focus on the cytopathology of PCL.

Our comparison of diagnostic yield strictly considered the pathology report, which was based on the impression of the pathologist from the cell types observed under the microscope as well as any appropriate staining used. For both FNA and FNB, if there was adequate fluid obtained, the fluid was evaluated for somatic genes and levels of glucose, amylase, and CEA to aid in clinical decision making, but they were not a source of determination in our study for diagnostic yield.

### Statistical analysis

We summarized and compared baseline participant and clinical characteristics between EUS-FNA and EUS-FNB using chi-square test for categoric variables, analysis of variance for continuous variables, and Wilcoxon signed-rank test for nonnormally distributed variables. The proportions of FNA versus FNB for determining the grade of IPMN cases were estimated with the use of the chi-square test and, for cells with frequencies <5, the Fisher test. A *P* value of <.05 was considered to be statistically significant. The relative risk (RR) was calculated by dividing the proportion of IPMN cases with an identified grade (both low-grade and high-grade dysplasia) in the FNB group by the proportion in the FNA group. All statistical analyses were performed using the R program (R Foundation for Statistical Computing, Vienna, Austria).

## Results

In this study, 130 cysts were identified. Of the 130 cysts, 34 were analyzed by means of FNA and 96 by means of FNB (Table 2). Of the 118 patients, 49% were female (Table 1). Overall, there were no statistical differences in the distribution of sex between FNA and FNB groups. The overall mean age of the patients was 68 ± 12 years old, with no significant difference in age between FNA and FNB groups. Patients in the FNB group had a significantly higher medical history for diabetes and vascular changes, which included aneurysms, coronary artery disease, deep vein thrombosis, and atherosclerosis (Table 1).

**Table 2:** Procedure and cyst characteristics as determined by EUS

	Overall (n = 130)	EUS type		*P* value†
		FNA (n = 34)	FNB (n = 96)	
Cyst size (mm)	24 ± 12	21 ± 8	25 ± 12	.058
Cyst location				
Head	46 (35)	12 (35)	34 (35)	>.9
Uncinate	18 (14)	2 (5.9)	16 (17)	.20
Neck	12 (9.2)	5 (15)	7 (7.3)	.30
Body	37 (28)	10 (29)	27 (28)	.90
Genu	4 (3.1)	1 (2.9)	3 (3.1)	>.9
Tail	30 (23)	5 (15)	25 (26)	.20
Pancreatic duct diameter, mm	2.60 ± 2.36	3.53 ± 2.94	2.35 ± 2.12	.023
Unknown	17	10	7	
Presence of …				
Septation	58 (45)	17 (50)	41 (43)	.50
Nodule	10 (7.7)	3 (8.8)	7 (7.3)	.70
Debris	13 (10)	0 (0)	13 (14)	.020
PD dilation	28 (22)	9 (26)	19 (20)	.40

Values are mean ± standard deviation or n (%).

*EUS,* Endoscopic ultrasound; *FNA,* fine-needle aspiration; *FNB,* fine-needle biopsy; *PD,* pancreatic duct.

† Fisher test; Wilcoxon rank sum test; Pearson chi-square test.

**Table 1:** Baseline clinical features

	Overall	EUS type		*P* value∗
		FNA	FNB	
Demographics	n = 118	n = 30	n = 88	
Sex				.3
Female		17 (57)	41 (47)	
Male		13 (43)	47 (53)	
Age at procedure, y	68 ± 12	67 ± 16	69 ± 11	.8
Medical history	n = 130	n = 34	n = 96	
Chronic pancreatitis	1 (0.8)	0 (0)	1 (1.0)	>.9
Vascular change	29 (22)	3 (8.8)	26 (27)	.03
Jaundice	3 (2.3)	2 (5.9)	1 (1.0)	.20
Weight loss	18 (14)	5 (15)	13 (14)	>.9
Diabetes	33 (25)	3 (8.8)	30 (31)	.01
Pain, abdominal/back	45 (35)	12 (35)	33 (34)	>.9
Pancreatitis	5 (3.8)	2 (5.9)	3 (3.1)	.60

Values are n (%) or mean ± standard deviation.

*EUS*, Endoscopic ultrasound; *FNA*, fine-needle aspiration; *FNB*, fine-needle biopsy.

∗ Wilcoxon rank sum test or Pearson chi-square test for demographics, Fisher test or Pearson chi-square test for medical history.

### Cyst characteristics as determined by EUS

Table 2 summarizes cyst characteristics as determined with the use of EUS before performing either FNA or FNB. Cyst size, reported as the largest cyst dimension, was not statistically different between the FNA and FNB groups. The location of the cyst, categorized by location in the head, uncinate, neck, body, genu, or tail of the pancreas, was similar between the FNA and FNB groups. The presence of septation, nodule, and pancreatic duct dilation was not significantly different. However, there was a statistically significant larger dilation of pancreatic duct diameter in the FNA group (*P* = .02) and significantly more necrotic debris detected in the FNB group (*P* = .02).

### Diagnostic outcomes

**Figure 1:**
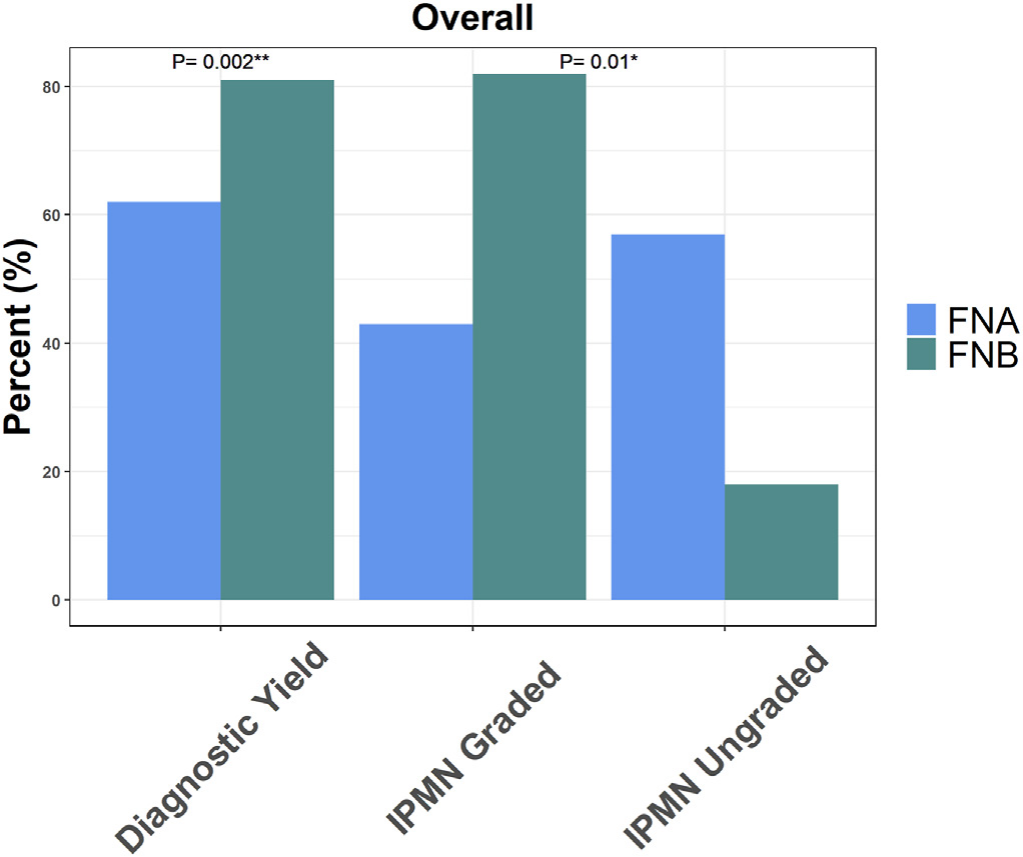
Overall yield. *FNA*, Fine-needle aspiration; *FNB*, fine-needle biopsy; *IPMN*, intraductal papillary mucinous neoplasm.

As summarized in Figure 1, the overall diagnostic yield was significantly higher with FNB than with FNA (81% vs 62%; *P* = .02), with FNB performing significantly better than FNA in identifying cyst grade for IPMN (RR = 1.92; *P* = .013).

Although not statistically significant, among the diagnoses, there was 1 rare case of lymphoepithelial cyst and 3 cases of neuroendocrine tumor (NET) diagnosed with FNB compared with 1 case of NET diagnosed with FNA (Table 3).

**Table 3:** Diagnostic results

	Overall (n = 130)	EUS type		*P* value∗
		FNA	FNB	
Diagnostic yield	99 (76)	21 (62)	78 (81)	.022
IPMN	59 (45)	14 (41)	45 (47)	.6
Comparison of IPMN groups				.013
Ungraded	16 (27)	8 (57)	8 (18)	
Graded	43 (73)	6 (43)	37 (82)	
Metastatic cancer	3 (2.3)	1 (2.9)	2 (2.1)	>.9
Inflammatory process	9 (6.9)	0 (0)	9 (9.4)	.11
SCA	15 (12)	1 (2.9)	14 (15)	.11
Sample not adequate/specimen insufficient for evaluation	7 (5.4)	3 (8.8)	4 (4.2)	.4
Nonmalignant sample	1 (0.8)	1 (2.9)	0 (0)	.3
Inconclusive features	23 (18)	9 (26)	14 (15)	.12

Values are n (%).

*EUS,* Endoscopic ultrasound; *FNA,* fine-needle aspiration; *FNB,* fine-needle biopsy; *IPMN,* intraductal papillary mucinous neoplasm; *SCA,* serous cystadenoma.

∗ Wilcoxon rank sum test, Pearson chi-square test, Fisher test.

### Adverse events

Neither procedure had immediate AEs such as bleeding, perforation, pancreatitis or infection, rehospitalization, emergency visit, or any other AE within the 7 days following the procedure.

### Discussion

Incidental PCL detection warrants further risk stratification of the cyst to guide management. Considering the high (20%-40%) morbidity risk of pancreatic surgery and the malignant potential of PCLs, choosing a diagnostic method that will yield the most diagnostic information for differentiating benign from premalignant and malignant cysts is essential.^2^

Although FNB can be used in pancreatic solid lesions, in our knowledge there currently exists little information regarding the use of FNB in PCLs. In our comparison of the diagnostic yield of EUS-FNA versus EUS-FNB for PCLs, we found that the overall diagnostic yield was significantly higher in FNB than in FNA (81% vs 62%; *P* = .02), with FNB performing significantly better than FNA in identifying cyst grade for IPMN (RR = 1.92; *P* = .013). This result is reflective of the main advantages of FNB: the preservation of cyst wall histologic architecture. In addition, our pathologists were able to test biopsy samples taken with FNB from the cyst wall for high-risk markers such as SMAD4 and TP53, which can further assist clinicians with risk stratification, surveillance, and surgical candidacy. Although not statistically significant, among the diagnoses there was 1 rare case of lymphoepithelial cyst and 3 cases of neuroendocrine tumor diagnosed with FNB, compared with 1 case of NET diagnosed with FNA. The sex, age, location, and presence of pancreatic ductal dilation were not significantly different between the FNA and FNB groups. Both procedures in our study were safe with no recorded AEs.

Limitations of this study include its retrospective nature, limited sample size, and involvement of trainees. Given the sample size, our results may be affected by type II error, and the retrospective and single-institutional nature of our study predisposes it to selection bias. On the other hand, the retrospective nature can mitigate the susceptibility to performance bias, a factor that could be present in randomized controlled trials involving procedures where blinding may not be possible. Because PCLs have a relatively low incidence and prevalence, one of the strengths of our study includes utilization of a high-volume comprehensive cancer center, board-certified advanced endoscopy-trained gastroenterologists, and cytopathologist with expertise in PCL cytology, all of which may pose limitations on making this study reproducible in a community hospital. Additional limitations include pathology reports obtained retrospectively having been analyzed by 1 pathologist.

In this study, cyst type was determined solely based on the cytology or histology as determined by pathology reports rather than relying on EUS, CT, or MRI findings and may not directly correlate to clinical risk for certain PCLs such as IPMNs. For example, a low-grade IPMN with a nodule in the main pancreatic duct may have a high clinical risk. Instead, our study focused on assessing the performance of 2 tissue acquisition techniques, because the use of EUS and imaging was a shared feature between the 2 techniques.

Using FNB for PCLs is a novel approach that has not been commonly described before for pancreatic cysts.^13^ With this approach, we were able to risk stratify more lesions to identify patients for surveillance and surgical candidacy. This is the first study in our knowledge to compare the diagnostic outcome of FNA and FNB for PCLs, so it is difficult to comment on our results in comparison to known literature. The results show promising data in the use of FNB for PCLs. Given the higher diagnostic yield, it is possible that with FNB a greater number of patients could receive a diagnosis with fewer procedures, leading to early management. In contrast, in cases of nonconclusive tissue samples, patients need to undergo repeated sampling, which increases the cost of health care and delays intervention.

Future studies in this area can aim to bridge the limitations of our study, for example, by designing a randomized controlled trial comparing the performance of FNA and FNB in PCLs. Considering the current lack of evidence-based guidelines for choosing between FNA and FNB, future research could also explore the relationship between cyst radiologic features and diagnostic outcomes for PCLs using either technique. This exploration would help inform clinical decisions regarding the choice between FNA and FNB for PCLs.

## Conclusion

Our results reflect one of the first studies, to our knowledge, to consider the diagnostic role of FNB in evaluating PCLs. These preliminary results suggest that FNB may have a potential for high diagnostic performance in PCLs and warrant further exploration using a larger cohort and randomized controlled trails.

## Disclosure

The following authors disclosed financial relationships: A.K. Luthra: consultant for Boston Scientific. S. Cappelle: consultant for Olympus. S.R.S. Mok: consultant for Conmed, Steris, and Pentax Medical (C2 CryoBalloon). All other authors disclosed no financial relationships.

## References

[1] Hu J.X., Zhao C.F., Chen W.B. (2021). Pancreatic cancer: a review of epidemiology, trend, and risk factors. World J Gastroenterol.

[2] Ardeshna D.R., Cao T., Rodgers B. (2022). Recent advances in the diagnostic evaluation of pancreatic cystic lesions. World J Gastroenterol.

[3] Scholten L., van Huijgevoort N.C.M., van Hooft J.E. (2018). Pancreatic cystic neoplasms: different types, different management, new guidelines. Visc Med.

[4] Elta G.H., Enestvedt B.K., Sauer B.G. (2018). ACG clinical guideline: diagnosis and management of pancreatic cysts. Gastroenterology.

[5] Tanaka M., Fernández-del Castillo C., Kamisawa T. (2017). Revisions of international consensus Fukuoka guidelines for the management of IPMN of the pancreas. Pancreatology.

[6] Facciorusso A., Wani S., Triantafyllou K. (2019). Comparative accuracy of needle sizes and designs for EUS tissue sampling of solid pancreatic masses: a network meta-analysis. Gastrointest Endosc.

[7] Thornton G.D., McPhail M.J.W., Nayagam S. (2013). Endoscopic ultrasound guided fine needle aspiration for the diagnosis of pancreatic cystic neoplasms: a meta-analysis. Pancreatology.

[8] Mohan B.P., Madhu D., Khan S.R. (2022). Intracystic glucose levels in differentiating mucinous from nonmucinous pancreatic cysts: a systematic review and meta-analysis. J Clin Gastroenterol.

[9] Levine I., Trindade A.J. (2021). Endoscopic ultrasound fine needle aspiration vs fine needle biopsy for pancreatic masses, subepithelial lesions, and lymph nodes. World J Gastroenterol.

[10] Facciorusso A., del Prete V., Antonino M. (2020). Diagnostic yield of EUS-guided through-the-needle biopsy in pancreatic cysts: a meta-analysis. Gastrointest Endosc.

[11] Cotton P.B., Eisen G.M., Aabakken L. (2010). A lexicon for endoscopic adverse events: report of an ASGE workshop. Gastrointest Endosc.

[12] Nagtegaal I.D., Odze R.D., Klimstra D. (2020). The 2019 WHO classification of tumours of the digestive system. Histopathology.

[13] Phan J., Dawson D., Sedarat A. (2020). Clinical utility of obtaining endoscopic ultrasound-guided fine-needle biopsies for histologic analyses of pancreatic cystic lesions. Gastroenterology.

